# Factors influencing biospecimen collection in decentralized pregnancy and birth cohorts: A qualitative study

**DOI:** 10.1017/cts.2025.10099

**Published:** 2025-07-30

**Authors:** Melissa Weidner, Nashwah Azam, Amulya Gobburu, Michelle Jansen, Zorimar Rivera-Núñez, Veenat Parmar, Gloria Bachmann, Nancy Reilly, Reynold Panettieri, Maria Gloria Dominguez-Bello, Martin J. Blaser, Daniel B. Horton, Emily S. Barrett, Slawa Rokicki

**Affiliations:** 1 Department of Pediatrics, Rutgers Robert Wood Johnson Medical School, New Brunswick, NJ, USA; 2 Department of Health Behavior, Society and Policy, Rutgers School of Public Health, Piscataway, NJ, USA; 3 Center for Advanced Biotechnology and Medicine, Rutgers University, Piscataway, NJ, USA; 4 Department of Biostatistics and Epidemiology, Rutgers School of Public Health, New Brunswick, NJ, USA; 5 Environmental and Occupational Health Sciences Institute, Rutgers University, Piscataway, NJ, USA; 6 Department of Obstetrics, Gynecology and Reproductive Sciences, Rutgers Robert Wood Johnson Medical School, New Brunswick, NJ, USA; 7 Rutgers Institute for Translational Medicine and Science, New Brunswick, NJ, USA; 8 Department of Medicine, Rutgers Robert Wood Johnson Medical School, New Brunswick, NJ, USA; 9 Department of Biochemistry and Microbiology, Rutgers University, New Brunswick, NJ, USA; 10 Department of Anthropology, Rutgers University, New Brunswick, NJ, USA; 11 Rutgers Center for Pharmacoepidemiology and Treatment Science, Institute for Health, Health Care Policy and Aging Research New Brunswick, NJ, USA

**Keywords:** Decentralized research, biospecimens, pregnancy and birth cohort, qualitative research, study design

## Abstract

**Introduction:**

Although decentralized research is being used more frequently, few data are available regarding barriers for potential subjects related to engaging in decentralized research with remote biospecimen collection, especially within pregnancy and birth cohorts that include individuals of diverse racial and ethnic backgrounds.

**Methods:**

Focus groups and individual interviews with pregnant and postpartum women were conducted in English and Spanish. Thematic analysis was used to identify motivators and barriers to participation in decentralized research involving biospecimens.

**Results:**

Sixty women (35% Hispanic/Latino, 23% Black, 18% Asian, 15% non-Hispanic White) participated in 10 focus groups (English = 8, Spanish = 2) and 11 individual interviews (English = 7, Spanish = 4). Three themes emerged about factors that could promote participation in decentralized biospecimen collection: 1) convenience, 2) autonomy, and 3) benefit (to self, community or society). Four themes emerged about potential barriers: 1) lack of interaction with trained professionals, 2) inability to coordinate with existing clinical care, 3) discomfort and invasiveness, and 4) concerns about data transparency and security. Overall, participants felt more comfortable providing biospecimens for themselves compared to their child and with biospecimens perceived as less painful or invasive to obtain.

**Discussion:**

Our findings suggest that transparency about the purposes and use of collecting biospecimen and clear instructions (such as written and instructional videos) could improve biospecimen collection in decentralized pregnancy and birth cohorts. Additionally, opportunities for virtual interaction with study staff and options related to collection of certain biospecimens such as blood (mobile collection unit with trained staff versus a self-collection device) may also improve participant engagement.

## Introduction

The widespread availability and use of the internet has created opportunities for research studies to recruit and engage participants without the need for onsite visits. Terms to describe research in which participant activities are not conducted at traditional onsite locations have included “decentralized,” “virtual” and “direct-to-participant.” [1] In a fully decentralized study, subject recruitment as well as data and biospecimen collection occur without in-person contact between the study team and participant [1]. Decentralized research has been applied in many ways including decentralized clinical trials, web-based medical devices, large-scale citizen science studies, disease-specific cohort studies and observational cohorts [1–9]. The focus of this manuscript is in regard to decentralized research involving digital data collection and “remote” biospecimen collection in which samples are self-collected and mailed in by the participants.

Decentralized research studies have several potential benefits to participants, including reducing financial and time burdens of participation as well as transportation-related barriers, while providing a greater sense of anonymity [1–3]. Given the broad reach of the internet, large-scale studies are currently being conducted to answer complex and important health-related research questions that include remote biospecimen collection [4–8]. Cohort studies have shown the feasibility of decentralized recruitment and enrollment, with remote biospecimen collection [4–9]. For example, the American Gut Consortium, a large participatory science project, has enrolled over 7,000 participants in the United States alone, with subjects providing stool samples and other data remotely [4].

However, decentralized research, especially studies that involve long-term commitment, have unique challenges with participant recruitment, engagement, and biospecimen collection. The Pre-Mace Study evaluated feasibility of remote dried blood sampling by comparing return rates and specimen quality of biospecimens from remote (at home) and in-clinic collection. The study found that rates of return were significantly higher in the in-clinic collections, although quality of biospecimens was comparable between the two groups [9]. Self-selection bias and lack of racial and ethnic diversity have also been challenges in decentralized research [7–8]. In the Human Epidemiology and Response to SARS-CoV-2 (HEROS) study, a decentralized prospective study of adults and children across 12 U.S. cities, high drop-out rates were observed, particularly among African-Americans [7]. High attrition may be due to participant burden, complicated biospecimen collection instructions, and limited opportunities for participants to interact with study staff. Similarly, while a pre-conception cohort study, PRESTO, has had considerable success with recruitment and biospecimen collection, recruiting diverse participants has been a challenge and the cohort largely identifies as White and well-educated [8].

Pregnancy and early childhood represent critical windows in the development of health and diseases [10]. Research has demonstrated that maternal and early life exposures are associated with increased risk of developing metabolic, atopic, and immune-mediated diseases [11–22]. Studies involving biospecimen collection can provide data crucial to understanding mechanistic links between exposure and disease risk [23–28]. Although decentralized studies can provide an alternative recruitment strategy for researchers, data regarding factors that contribute to successful recruitment and engagement is limited, especially related to birth cohorts. To our knowledge, one study has examined the unique motivators for and barriers to remote biospecimen collection in pregnancy or birth cohorts [29]. Lemas et al. conducted semi-structured interviews of pregnant and breastfeeding women regarding engagement in longitudinal studies involving non-invasive biospecimen collection in pregnancy and birth cohorts. Participants raised concerns about stool collection in pregnancy (given constipation is common in pregnancy) as well as breastmilk collection (citing concerns about volumes needed and potential for low breastmilk supply) [29]. Participants spoke favorably about the convenience of dropping-off remotely collected biospecimens at local sites [29]. Although Lemas et al. acknowledged a considerable gap in knowledge regarding factors associated with engagement in decentralized studies, there are limitations in the generalizability of their findings given that their participants tended to be well-educated women in their 30 – 40s with prior experience in research participation [29].

To inform best practices in developing and implementing a longitudinal birth cohort study with decentralized recruitment and remote biospecimen collection, we conducted focus groups and individual interviews with pregnant and postpartum women. Our primary aim was to understand the potential advantages and barriers to participation in decentralized research involving remote biospecimen collection during and shortly after pregnancy. We were particularly interested in understanding the factors associated with participation among underrepresented populations as previous decentralized cohort studies have struggled with enrollment and retention of socio-demographically diverse participants.

## Methods

This study used an exploratory qualitative study design. Participants were recruited in person at obstetric care clinics in two large hospitals located in northern and central New Jersey as well as at community events during the fall of 2022. Participants were also recruited through the social media platforms of several trusted community-based organizations. Individuals expressed interest by completing an online form with their contact information or by directly calling or emailing the study team from information posted on a flyer. These volunteers were then called and screened, and eligible participants were scheduled for a focus group or individual interview. Eligibility criteria for participation included: at least 18 years of age, currently pregnant or less than 1 year postpartum, current resident of New Jersey, and speaks either English or Spanish.

Focus groups, conducted on Zoom, allowed participants to interact and expand viewpoints in context with others’ contributions. In person individual interviews were used to enhance recruitment for participants who could not commit to a scheduled Zoom focus group but were willing to participate before or after their prenatal appointment. Participants recruited from obstetric clinics were provided the option between an individual in-person interview that day (before or after the patient’s prenatal appointment) or a scheduled focus group discussion via Zoom. Those recruited via other forms of recruitment (social media of community-based organizations or community events) were scheduled for focus group interviews via Zoom since recruitment was conducted throughout the state.

Focus groups lasted 1 hour, while individual interviews lasted 20 minutes. Focus groups and individual interviews were conducted in English or Spanish and were recorded, translated, and transcribed by trained study staff. Data were fully de-identified; therefore, individual participant responses were not associated with independent study identification numbers.

Focus groups and individual interviews were conducted between August 1, 2022 and November 4, 2022 by MJ, NA, a research coordinator and two students supervised by MJ. Interviewers had Master’s degrees or were Master’s students trained in qualitative interviewing methods. The research coordinator is a native Spanish speaker who conducted Spanish interviews/focus groups.

The questions used in the focus groups and interviews were developed based on prior research on factors that promote participant engagement in decentralized research [30–31]. Questions were intended to elicit feedback from participants about their level of comfort with participating in a research study during pregnancy, providing biospecimens for themselves and their children, preferred modes of collection for biospecimens, and motivators for and barriers to participation. Transcripts were analyzed using a general thematic approach through an iterative process of coding and discussion [32]. Analysis was guided by a framework developed by Killien et al. regarding potential barriers and motivating factors impacting participation in clinical trials [33].

The analysis was conducted by four trained coders, (SR, AG, NA, and MR) (all women including the PhD-level primary investigator, two Master’s level researchers, and one Master’s student), with each transcript independently reviewed by at least two coders.

After developing an initial codebook based on the interview guide, a subset of transcripts were coded by all four coders and the codebook was refined. Coding was then conducted for all recorded transcripts. Coders met weekly to review and refine coding definitions through discussions of coded transcripts. When coding was completed, all coders met to collaboratively identify emergent themes and discuss any conflicting results until consensus was reached [32]. Data were analyzed using NVivo [35]. For the current analysis, we focused on responses that were specifically related to biospecimen collection.

## Results

A total of 100 participants were screened, 60 of whom completed a focus group or individual interview (Appendix A: flow diagram of participant selection process). Ten focus groups were empaneled (each with 2 – 8 participants) and 11 individual interviews were completed (Table 1). Two focus groups and 4 individual interviews were conducted in Spanish, while the remainder were conducted in English.

**Table 1.** Referral source and interview type

Referral source [*n* (%)]	Focus group *n* = 49	Individual interview *n* = 11	Total *n* = 60
Hospital-based obstetric clinic #1	16 (33%)	6 (54%)	22 (37%)
Hospital-based obstetric clinic #2	6 (12%)	4 (37%)	10 (17%)
Family Festival	9 (18%)	1 (9%)	10 (17%)
Baby Expo	4 (8%)	0	4 (7%)
Word of mouth	7 (14%)	0	7 (12%)
Facebook	4 (8%)	0	4 (7%)
Other/not sure	3 (6%)	0	3 (5%)

The average age of participants was 32.7 years (range 21– 43) (Table 2). All participants identified as female. With regard to race and ethnicity, one-third (35%) identified as Hispanic or Latino, 23% were non-Hispanic Black, 18% were non-Hispanic Asian, 15% were non-Hispanic White, and 7% were more than one race. There was wide distribution across education levels (Table 2). There were differences in demographic characteristics among participants in the focus groups compared to those who completed individual interviews. Participants in individual interviews were more likely to identify as Hispanic/Latino and had higher education levels than those who participated in focus groups, although the number of individual interviews was small.

**Table 2.** Participant demographic and interview characteristics

Characteristic	All participants *n* = 60	Focus group participants *n* = 49	Individual interview participants *n* = 11
**Age in years** [mean (range)]	32.7 (21 – 43)	32.8 (21 – 43)	32.1 (23 – 42)
**Language of interview** [*n* (%)]			
English	49 (82%)	42 (86%)	7 (64%)
Spanish	11 (18%)	7 (14%)	4 (36%)
**Race/Ethnicity**			
Hispanic/Latino	21 (35%)	16 (33%)	5 (45%)
Non-Hispanic white	9 (15%)	6 (12%)	3 (27%)
Non-Hispanic black	14 (23%)	13 (27%)	1 (9%)
Asian	11 (18%)	11 (22%)	0
More than one race/other	4 (7%)	3 (6%)	1 (9%)
Missing	1 (2%)	0	1 (9%)
**Highest level of education** [*n* (%)]			
High School/Vocational trade school	11 (18%)	10 (20%)	1 (13%)
Associate’s degree	6 (10%)	6 (12%)	0
Some college	14 (23%)	9 (18%)	5 (45%)
College degree	14 (23%)	12 (24%)	2 (18%)
Graduate degree	14 (23%)	12 (24%)	2 (18%)
Missing	1 (2%)	0	1 (9%)

Description of participant’s reported demographic characteristics such as age, race/ethnicity and highest level of education. Also included is the language in which individual interviews or focus groups were conducted. N indicates the number of participants.

Overall, themes from the focus groups and interviews were classified into two broad categories related to participation in decentralized research involving remote biospecimen collection during pregnancy and the postpartum period: 1) potential benefits and 2) potential barriers.

### Potential benefits to remote biospecimen collection

Three themes emerged about potential benefits of participation in decentralized research involving remote biospecimen collection during and after pregnancy: 1) convenience, 2) autonomy and 3) benefit (to self, community and society) (Appendix B: table of representative quotes from participants regarding reported benefits of remote biospecimen collection).

#### Convenience

Many participants described the potential benefit of convenience in decentralized research related to factors such as time and not needing childcare and transportation. *“… I think having a kit and collecting it myself would be preferable. It eliminates the other barrier, right? Having to go somewhere and having another person administer it*.” Convenience of remote biospecimen collection was mentioned as a motivating factor for collection in children. *“I think that I would be more motivated if there was a home kit for swabbing like the rapid COVID test or something. You have them [children] at home and it would be something convenient…”*

The frequency of collection was also discussed in several focus groups and individual interviews. Especially for longitudinal studies in which participants are expected to remain engaged over time, less frequent collections, some even suggested yearly, were more appealing. Clear instructions were also cited as important for remote biospecimen collection to be convenient and successful, “*Mailing [biospecimens] is definitely convenient…I would feel comfortable as long as there’s instructions. .* .” Transportation barriers were raised by several participants, “*The problem is I don‘t work, I don‘t drive actually… So there’s no chance to drive to go to any hospital or any place to give to the sample or something like this*.” Another woman stated, “[The barrier] *would be the transport, if the study is conducted far away. Some of us don‘t know how to drive*.”

#### Autonomy

Some participants reported increased psychological comfort with remote biospecimen collection as this can provide a greater sense of autonomy related to self-collection of biospecimens. This was particularly important in the collection of biospecimens that were perceived as more sensitive or private, which in our cohort included vaginal swabs. “[*Regarding a vaginal swab] I already don‘t like having to do [it at] my regular checkups. But if it were something that if it were possible where it was a self-swab… if it were a more sensitive area, that would make me more inclined to be willing to do it*.” For many, autonomy of self-collection was preferred, but again the importance of clear instructions was mentioned by participants. “*As long as I have clear instructions on how to do it, I’d be more comfortable doing it myself.”*

#### Benefit to self, community, or society

Many participants reported benefits to themselves, their community, or society at large as motivation to participate in decentralized research involving a pregnancy or birth cohort. “*I would love to be part of the study to know that I could help future kids or moms in some way*… *because I’m really big on impact and things being beneficial to the community because I’m a community advocate. It would help me commit more if I knew that the studies that we were doing was going to benefit people in a large way*.” Other participants thought that personal benefit could be a motivating factor for participation, such as through resource guides, parental education, or returned information on personal health results. Multiple participants felt resources or education would be particularly helpful for first-time mothers. *“Especially the newer moms because they‘re not aware of all the resources and things… So just to be able to make an awareness or have a platform for them could be very helpful.”* One woman reported that she would be interested in participating in research about a specific health condition that she has, to benefit others with this condition. “*I have Graves’ disease. …. So this pregnancy has been rough for me … My story might be able to help somebody else that might be going through thyroid or Graves’ disease or something like that.”* Although direct compensation was not considered the *“primary motivating factor,”* some women reported that direct compensation such as coffee or a gift card would be a motivating factor.

#### Potential barriers to remote biospecimen collection

Four themes emerged about potential barriers to engagement in remote research involving biospecimen collection during and after pregnancy: 1) lack of interaction with trained professionals, 2) inability to coordinate with existing clinical care, 3) discomfort and invasiveness and 4) concerns about data transparency and security (Appendix C: table of representative quotes from participants regarding reported barriers of remote biospecimen collection).

#### Lack of interaction with trained professionals

Although many women reported greater convenience and autonomy of self-collected biospecimens at home, for some women self-collection was not preferred. Concerns about collection of biospecimens without a trained professional present ranged from concerns about the actual collection to proper processing and shipping. Some participants had concern regarding the entire range of the remote biospecimen process, “*as long as a professional doctor is doing it. I do not want to be having to collecting samples and shipping them to you and things like that.*” While others had more focused concerns, “*I‘m the type of person that would take a sample and forget about it leaving on my dresser and be like, oh I‘m supposed to mail this and by the time you guys get it it’s like, oh well, we could not use the sample.”* There was less acceptance of self-collection of biospecimens on children, examples included, “*I prefer a health [care] provider to be assisting and taking all those samples from my baby.*” and “*I think for myself, a kit on my own is fine. But for my child, I think I would prefer a professional*.” For other women, trained staff was preferred for collection of certain types of biospecimens such as blood, *“… but for the blood drawn and stuff, a professional … actual certified nurse practitioner or a doctor [would be preferred].*” Many women had concerns about their ability to successfully and accurately collect biospecimens from their child. One participant stated, “*I’m not normally the person who holds the kid down*.” Another woman responded, “*For my eight year old son, any time I‘ve had to do a COVID test on him, a home one, I do four, because I‘m so terrified that I‘m like, “I didn‘t get enough,” and like I torture him. So I would be so scared that I didn‘t do it properly. I would need a professional.”* The importance of clear instructions for biospecimen self-collection was again noted by several women. “*If there were good instructions, I would be comfortable attempting it [biospecimen collection] myself …”*

#### Inability to coordinate with existing clinical care

Traditional in-person studies are often conducted at a clinic or hospital, which may allow for coordination with existing medical care. Some women reported concerns about the additional “steps” of being involved in decentralized research. *“I would be more inclined also if the research could be done while I was already going to see the OB or while my kids already had an appointment scheduled and it could be done while we were waiting for the doctor or something.”* Other women preferred that biospecimens obtained could “*be utilized for both the purpose of whatever their routine medical appointment was and could be utilized for this study*” or “*replace”* recommended clinical testing and therefore reduce some of their costs associated with healthcare.

#### Discomfort and invasiveness

The most reported potential barriers to remote participation and biospecimen collection related to discomfort and invasiveness. However, there was substantial individual variation about which types of specimens were deemed uncomfortable or invasive. Most women seemed comfortable with providing saliva, nasal and skin swabs as well as stool and urine samples from themselves. However, many women reported hesitation about blood specimens, and some women had hesitation about vaginal swabs. As discussed above, some women were comfortable with swabs from more sensitive locations such as vaginal swabs as long as they were self-collected, whereas others were not, “*I do not know about vaginal swabs during pregnancy, but the other samples I think I would be fine with*.” It is unclear if women who were uncomfortable with vaginal swabs during pregnancy were concerned about risk to the baby or had other concerns. Given that pregnancy can be associated with discomfort, some women were less agreeable to collecting biospecimens they deemed as invasive while pregnant. *“It’s just like I‘ve been really miserable this whole pregnancy… as long as it’s not invasive, then I‘m fine.”*

In general, women reported more concerns about obtaining biospecimens from their children than from themselves. The spectrum of comfort related to collecting biospecimens on their child ranged from discomfort with any type of biospecimen to discomfort with certain specimens perceived as more uncomfortable or invasive. Some women were uncomfortable with any biospecimen collection on their child during infancy regardless of the method of collection- traditional in-clinic vs remote self-collection. One woman stated, “*Honestly, I wouldn‘t feel comfortable with you at all taking samples from my baby.” Another participant reported, “I just don‘t want to do that to my baby. That’s all*.” Blood samples from children were a particular concern for several women, “*I’m just very uncomfortable about providing blood samples. I just don‘t want anything like those needles to go into my baby’s body…” and “Yeah, I don‘t have any issue providing any samples for myself, but for my baby swabs? Yes, but not the blood samples*.” Although biospecimens perceived as coming from more “private” body areas were also discussed, *“If I really wanted to convince myself for my child, I think I would be okay with everything else except for urine and feces just because it has to deal with their private parts.”*

There were also concerns and questions raised about how certain specimens would be obtained. “*How are they going to get that microbiome specimen from the skin of the baby? Are they scraping the skin or how are they going to do that?”* Transparency regarding the reasons that each biospecimen is being collected, especially for biospecimens from children were important to many women. “*I‘d wanna really look at all the different [biospecimen types], the reasons for each type of sample collection.”* Many women had concerns about collecting biospecimens from their child, especially in terms of age and size of the child, having comments such as “. *. . She’s just too small.*” and “*My kids are already scared of the doctor’s office when they’re getting shots every six months and so if they’re older, also more inclined to do it*.”

Concerns about autonomy and the ability of the child to provide assent/consent were also raised related to biospecimen collection in general. “*Just my child being touched in areas that they didn‘t ask for. They didn‘t ask to join this.”* However this participant’s concerns were in relation to traditional in-person biospecimen collection and it is unclear if this type of concern would be lessened through remote biospecimen collection (parental or self-collection). *“I don‘t know, I just feel it would make me super uncomfortable to bring my child somewhere to say, oh, you have to do this… I don‘t think I can bring myself to do that for research. It doesn‘t make me comfortable in that way*.”

#### Concerns about data transparency and security:

Participants raised concerns about data transparency and security, especially in regard to biospecimens containing DNA. Transparency of how data would be used and shared was important to most women. *“I would like to know what all that would be done with these samples.”* Several women had concerns about providing samples for research involving DNA. One woman had questions regarding DNA use and confidentiality such as, “*I want to know how is that [genetic] material used? How is it stored? Would it be discarded? The confidentiality around genetic data*?” Another woman reported concerns about data security and transparency especially regarding DNA, stating, “… *it’s so easy to have information, not be private or have security risks these days and also have it initially be for one purpose and then used for another. I would just be very wary of what it means if you were able to get DNA blood information*.”

## Discussion

Given the paucity of literature on decentralized research in pregnant women and their children, this study provides insights into perceived benefits and barriers to participant engagement with a focus on remote biospecimen collection.

After qualitative analysis from responses of participants in our study, four themes emerged regarding barriers to remote biospecimen collection. Table 3 summarizes these barriers and five proposed potential solutions which address all barriers identified by our cohort. Instructional videos can be tailored to address all of the identified barriers and can be available to participants in various phases of engagement including at recruitment as well as throughout the study. Optional virtual interactions with study team members can also address multiple of the identified barriers including lack of interaction with trained professionals, discomfort and invasiveness and concerns about data transparency and security. Finally, for certain types of biospecimens, we propose several potential solutions including 1) multiple collection options (for example mobile collection units vs self-collection device for blood), 2) opt-in or opt-out options and 3) additional incentives for opt-in biospecimens.

**Table 3.** Barriers for decentralized biospecimen collection and proposed solutions

Barrier	Proposed solutions
Lack of trained professional	Instructional videos demonstrating collection techniques as easy, quick and painless Optional virtual interactions with study team members to address questions regarding biospecimen collection technique or mailing
Inability to coordinate with clinical care	Emphasize benefits of convenience and autonomy provided through remotely conducted research through online introductory videos in recruitment materials
Discomfort and invasiveness	Instructional videos demonstrating collection techniques as easy, quick and painless Optional virtual interactions with study team members to address concerns regarding biospecimen collection For certain biospecimens deemed as “invasive” by participants consider the following: Various collection options- mobile collection unit vs self-collection Opt-in or opt-out options Additional incentives for opt-in biospecimens
Concerns about data transparency and security	Proactive recruitment materials including introductory videos in which study team members explain what biospecimens will be collected, why these are being collected and how they will be used/shared Optional virtual interactions with study team members to address concerns regarding data security

Some key findings from the participants in our study included greater comfort in providing biospecimens both perceived as less invasive and those obtained from the caregiver themselves compared to from their child, as well as the importance of transparency about data use and clear instructions for biospecimen collection. Overall, participants favored a decentralized study approach compared to traditional in-person studies, given the increased convenience and autonomy, particularly with regard to maternal biospecimen collection. As discussed above, the potential benefits of convenience and autonomy of decentralized research could be emphasized in recruitment materials such as online videos to encourage participant enrollment. This strategy may help alleviate barriers such as the inability to coordinate decentralized research with clinical care.

Participants’ concerns regarding providing biospecimens from their children were related to lack of child autonomy, concerns about discomfort and invasiveness of the collection and concerns about the caregiver’s ability to appropriately and adequately collect samples on their child. Based on responses from participants in our study, clear instructions and instructional videos demonstrating the collection process as painless, quick, and easy could help address these concerns and improve the willingness to provide biospecimens from children.

In general, there was also less comfort regarding biospecimens that were perceived as invasive or uncomfortable, although opinions on which types of specimens were deemed “invasive” varied among the participants. For example, some participants were not comfortable providing vaginal swabs, especially during pregnancy, while others seemed very comfortable as long as the vaginal swabs were self-collected. During three interviews, women asked for clarification on how skin swabs were obtained, initially having concern that this was painful, with one participant asking if this would involve “scraping” the skin. However, after explanation that a skin swab involves a light brushing of the skin’s surface with a cotton swab, each participant reported comfort with providing a skin swab. This suggests the importance of instructional videos that demonstrate proper collection techniques as well as the availability of study team members to virtually discuss biospecimen collection concerns. These practices could reduce hesitancy and misconceptions about biospecimen collection, resulting in improved rates of return.

In particular, the biospecimen that participants most often reported they would decline as self-collection, was blood. Although some participants were comfortable with providing blood samples collected from a trained professional, overall, participants reported concerns with the self-collection of blood. However, a limitation of the study was that the interview guide did not include a clear description or picture of self-collection blood devices which studies have shown can be painless, quick and easy such as the TAP® micro and Tasso+ ® [36,37]. Further research is needed to evaluate whether clear instructions and step-by-step videos on the self-collection of blood could relieve concerns about discomfort and the difficulty of remote collection. However, for certain biospecimens, such as blood, providing opt-in or opt-out choices or even options regarding mode of collection (self-collection device vs. mobile collection unit with trained phlebotomist) may improve rates of biospecimen collection. Additionally, given that the majority of participants agreed that financial incentives were a motivating factor for participation, providing additional incentives for blood self-collection may also improve the rate of return.

Finally, this study suggests addressing concerns about data transparency and security are crucial when designing remotely conducted studies that engage pregnant participants. Participants raised concern about various aspects of data transparency and security such as implications of DNA collection and sharing. Prior studies have raised concern about genomics data sharing, particularly the potential for discrimination within insurance coverage and employment opportunities [38]. Research has shown that transparency in DNA sharing is highly valued by potential research participants, emphasizing the importance of participant clarity on who can access and use their genetic information [39]. Proactively addressing concerns about data transparency and security may be even more important in recruitment for decentralized research where there is no familiarity or engendered trust with the study staff team. Recruitment materials could include online videos that explain what is being collected and why and how genetic data will be used and shared. In addition, optional interactions with study staff through virtual meetings or phone conversations could allow participants to further discuss these concerns.

One major strength of this study was that the perspectives from a diverse population of pregnant and recently pregnant individuals were included in terms of age, race, ethnicity and education level, in contrast to most prior work in this area. Although Lemas et al. studied factors that impacted biospecimen collection in pregnancy cohorts, the data was not focused on factors related to remote biospecimen collection. This is where this study adds additional insight into specific facilitators and barriers. Some participant responses from Lemas et al, favored research activities to be conducted in a “legitimate” location such as their physician’s office [29]. Although some participants in this study preferred trained medical professionals to collect biospecimens, women did not voice general concern about the legitimacy of decentralized research. This may be related to improved comfort with decentralized research as well as potential geographic or demographic differences between the two study populations.

One limitation of this study is that participant responses were de-identified, therefore no conclusions about perspectives based on sociodemographic representation could be made. Also, similar to the Lemas study, this cohort was somewhat better educated than the average US woman based on 2021 census data, however differences were small (39% of US women hold a bachelor’s degree or higher based on census data vs 46% of women in our cohort) [40]. However, to ensure a racially and ethnically diverse sample of participants, we employed a variety of recruitment strategies. Participants were recruited in-person from obstetric care clinics at two large hospitals, both serving large proportions of Black and Hispanic women. In addition, we recruited in-person at community events and via a flyer distributed by social media of a wide range of health and social organizations such as Women, Infants and Children (WIC) centers, federally qualified health centers, and community-based programs or family services. Participants in this study were from New Jersey and although the group was relatively diverse in terms of age, race and ethnicity as discussed above, the generalizability cannot be determined.

Another potential limitation of the study is that individual interviews and focus groups were conversational, meaning that participants were free to discuss and respond to any or all of the biospecimen types. Given participants were not probed on each biospecimen type, this interview style may limit available data. Finally, the majority of the participants for this study were recruited through traditional onsite methods (Table 1) and therefore this cohort’s experience in decentralized research maybe limited. Overall, through this qualitative research, motivating factors and barriers to participation in decentralized pregnancy cohorts with remote biospecimen collection were identified along with potential strategies to improve study engagement and sample collection.

## Conclusions

Many factors must be considered when designing decentralized pregnancy cohort studies. There may not be a “one size fits all approach” and further research about remote biospecimen collection options, particularly for blood (mobile collection unit vs self-collection device) needs to be explored. These findings suggest that participant education through clear instructions (including written and instructional videos) and transparency about data use and sharing with a focus on genomic data, may improve participation and biospecimen collection. Optional virtual interactions with study staff may also be helpful, although further study is needed. Additional research will be required to better understand the factors associated with successful recruitment, engagement and retention of diverse participants in decentralized pregnancy and birth cohort studies.

## Supporting information

10.1017/cts.2025.10099.sm001 Weidner et al. supplementary material Weidner et al. supplementary material

## Supplementary material

The supplementary material for this article can be found at https://doi.org/10.1017/cts.2025.10099.
