# Supplementary material for: Factors influencing biospecimen collection in decentralized pregnancy and birth cohorts: A qualitative study
Source: J Clin Transl Sci. 2025 Jul 30;9(1):e189. doi: 10.1017/cts.2025.10099 (PMC12505239; doi:10.1017/cts.2025.10099)
Supplement: Weidner et al. supplementary material [file S205986612510099Xsup001.docx]

**Supplemental Material**

**Appendix A:**

**Figure 1. Participant Selection Diagram**


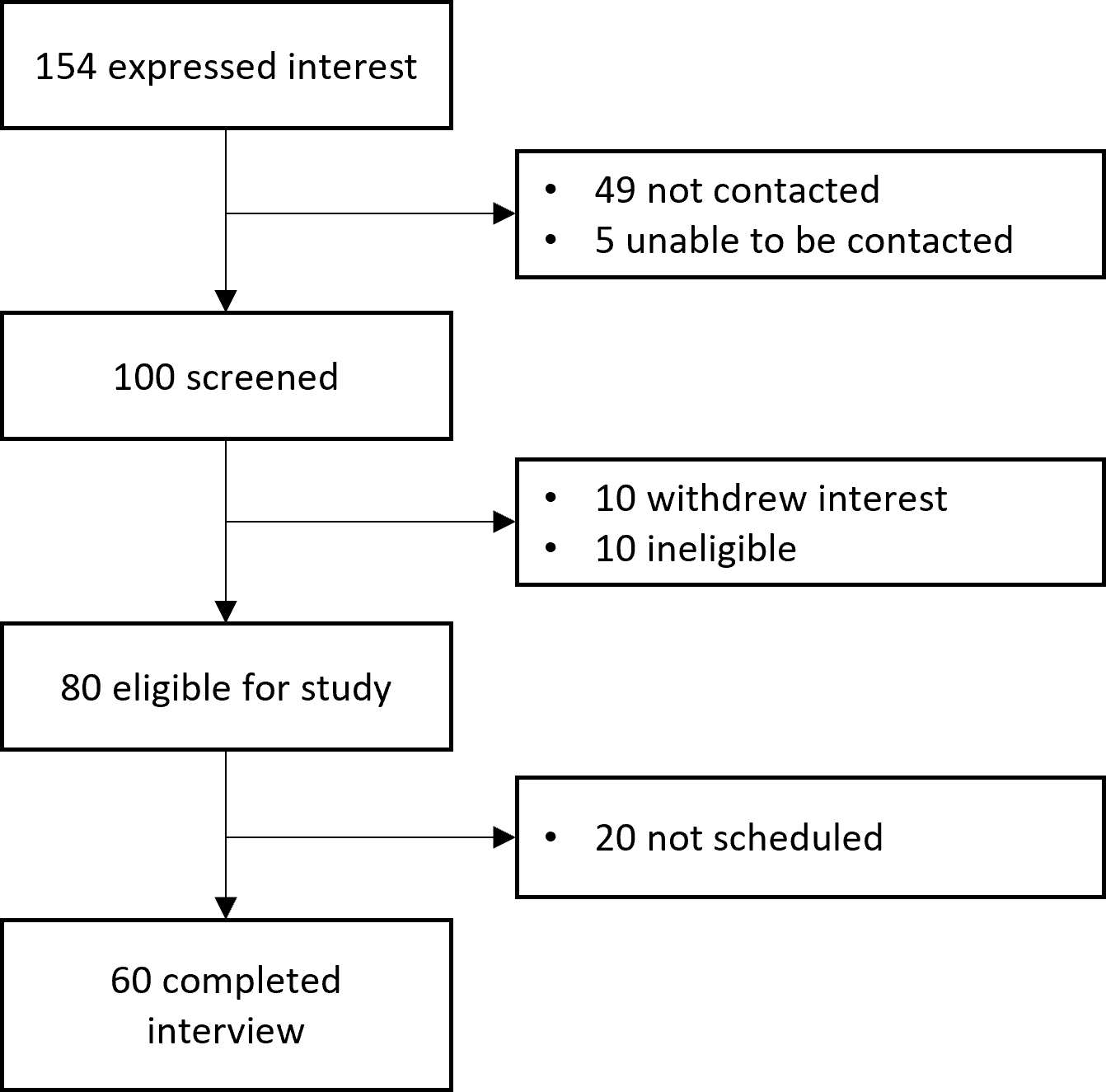


**Appendix B: Participant-reported potential benefits of remote biospecimen collection**

| Theme | Participant Quotations |
| --- | --- |
| **Convenience** | Mailing [biospecimens] is definitely convenient…I would feel comfortable as long as there’s instructions and things like that. I think I would be comfortable with that.  . . . But if you guys make it convenient for me, I say why not to do a study. . .  I think that it would be more motivated if there was a home kit for swabbing like the rapid COVID test or something. You have them at home and it would be something convenient and not as, I guess traumatizing or invasive to have to prepare the kid for because their home with you. The whole blood draw thing, I don’t know, I don’t even know how often I would keep up with that or how often you’d want to have your kids do that. But I do agree, that I think convenience and kids at home would make a difference as well.  As long as I’m not having to collect my blood sample or my baby’s blood sample. The swabs and saliva all that, I think having a kit and collecting it myself would be preferable. It eliminates the other barrier, right? Having to go somewhere and having another person administer it.  The problem is I don’t work, I don’t drive actually. So my husband only drives. So there’s no chance to drive to go to any hospital or any place to give to the sample or something like this.  [The barrier] would be the transport, if the study is conducted far away. Some of us don’t know how to drive. |
| **Autonomy** | As long as I have clear instructions on how to do it, I’d be more comfortable doing it myself.  When you were first explaining when you said vaginal swab, I was like, oh, I already don’t like having to do my regular checkups. But if it were something that if it were possible where it was a self-swab, the participant or the patient would be able to swab themselves if it were a more sensitive area, that would make me more inclined to be willing to do it. |
| **Benefit to the self, community and society** | I would love to be part of the study to know that I could help future kids or moms in some way. So I just mean I think if I knew what they were studying and I knew what difference could be made, I think that that would be motivating enough just for me at least.  If I’m able to contribute to this for the betterment of the larger group knowing that is good enough for me to be able to participate in such studies.  The biggest thing for me would be, what does the overall study does? Would this study benefit others? Would this make a change in research? Would this be something groundbreaking or impactful? Those are the things that would make me give more of a commitment because I’m really big on impact and things being beneficial to the community because I’m a community advocate. It would help me commit more if I knew that the studies that we were doing was going to benefit people in a large way.  I know that science advances a lot year after year and this is because studies like these are done and if they could help my baby and myself, yes I would do it, so that this can help future generation or my future children.  Especially the newer moms because they're not aware of all the resources and things that they know because some could be very young, some can be single parents, some cannot have family and they’re scared. So just to be able to make an awareness or have a platform for them could be very helpful because there's things that I didn't know when I was a first-time parent that I wish that I would have known. So just to be able to make a difference and try to help somebody else, that would be good.  So if you were taking my blood, and if you were looking at it just, I don’t know whatever you were looking at, I feel like I would like to know is something wrong or even like and everything’s okay, blood’s okay. Something like that would be nice. Especially since I think as a mom with small children, you don’t regularly get to the doctor. So if we did get to the doctor to have blood taken for this, it would sort of be a reassurance that everything was okay. It would serve as a medical visit place in my mind.  I have Grave [Graves’] disease. .... So this pregnancy has been rough for me . . . My story might be able to help somebody else that might be going through thyroid or Grave disease or something like that.  . . . Especially if it's going to help me and the baby in the long run. . .  Compensation is always appealing, I think that's a big motivator  If you paid for . . . I don't know, a coffee or something.  Well, a nice little incentive would be awesome. You know like the $50 gift card . . . [could be used towards] diapers.  [Compensation] might not be the primary motivating factor, but it could motivate, yeah… |

**Appendix C: Participant-reported potential barriers of remote biospecimen collection**

| Theme | Participant Quotations |
| --- | --- |
| **Lack of interaction with trained professional** | Well, I would prefer a kit at home for the vagina stuff, but blood drawn and stuff, a professional but not a data collector or a researcher but an actual certified nurse practitioner or a doctor.  Definitely doing it in the study center setting, often there’s two people helping hold down the kid which helps a lot, because the pediatrician’s office, they’re like, “I’m going to do this thing to your kid. You hold them down.” I’m like, “I’m not normally the person who holds the kid down.”  As long as a professional doctor is doing it. I don't want to be having to collecting samples and shipping them to you and things like that. . .  And I'm the type of person that would take a sample and forget about it leaving on my dresser and be like, oh I'm supposed to mail this and by the time you guys get it it's like, oh well, we couldn't use the sample.  I think for myself, a kit on my own is fine. But for my child, I think I would prefer a professional.  I prefer a health provider to be assisting and taking all those samples from my baby.  For my eight year old son, any time I've had to do a COVID test on him, a home one, I do four, because I'm so terrified that I'm like, "I didn't get enough," and like I torture him. So I would be so scared that I didn't do it properly. I would need a professional.  . . . I want to make sure that whoever is taking their [the child’s] specimen and samples is skilled in terms of taking care of children and taking their blood samples and stuff, because I've had really terrible experiences with my kids going to the doctor and to like [brand lab name redacted] or all these other places to take their blood. When people are not skilled, especially the little kids, their veins are so tiny, it really is traumatizing for them and it's traumatizing for parents to witness that. . .  . . . I probably wouldn't feel comfortable doing blood samples [self-collection] like other kinds of samples [insinuating would be comfortable with self-collection of samples other than blood], but as long as there's clear instructions about how everything is supposed to be done. |
| **Inability to coordinate with existing clinical care** | One thing I want to add is convenience. It already is an extra step usually to go to the doctor, to go to a lab and so I would be more inclined also if the research could be done while I was already going to see the OB or while my kids already had an appointment scheduled and it could be done while we were waiting for the doctor or something.  Could this testing replace any genetic testing that's being done so far? Could this be a benefit, because it would be free genetic testing versus the genetic testing we had to do in the doctor's office? That would be very motivating. Health care is really expensive, so if there was something tied into that where, I don't know, just regular  routine visits where then somehow I didn't have to pay as much, then that would be an added benefit and a motivating factor for the study. So even if it's not direct compensation but things that help to alleviate some of the stress of healthcare.  . . . If it was stuff like maybe for their routine medical appointments, certain like if they were having blood draws and that could be utilized for both the purpose of whatever their routine medical appointment was and could be utilized for this study, I think I would be more comfortable with that. |
| **Discomfort and Invasiveness** | . . . From myself, maybe certain swabs from areas. I don’t feel comfortable with you taking vaginal swabs for me. I don’t know.  For me, I think I would be comfortable with some level of that [biospecimen collection]. I don't know about vaginal swabs during pregnancy, but the other samples I think I would be fine with. They take enough blood from you when you're pregnant anyway.  It's just like I've been really miserable this whole pregnancy. But when I get pregnant, I'm always miserable. Like I said, as long as it's not invasive, then I'm fine. . .  As long as I’m not having to collect my blood sample or my baby’s blood sample. The swabs and saliva all that, I think having a kit and collecting it myself would be preferable. . .  I don't know about vaginal swabs during pregnancy, but the other samples I think I would be fine with. They take enough blood from you when you're pregnant anyway.  How are they going to get that microbiome specimen from the skin of the baby? Are they scraping the skin or how are they going to do that?  I’m just very uncomfortable about providing blood samples. I just don’t want anything like those needles to go into my baby’s body. She’s just too small.  . . . My kids are already scared of the doctor’s office when they’re getting shots every six months and so if they’re older, also more inclined to do it.  Yeah, I don’t have any issue providing any samples for myself, but for my baby swabs? Yes, but not the blood samples.  Any kind of personal test, I would do it. But for my baby, it’s like I get very sentimental about it. If it’s a urine test, that’s okay, but doing a blood test on my baby is tricky for me. It’s hard for me to take my children for vaccination. I get scared. In fact, my husband handles those situations. So I would be a little hesitant regarding my baby.  If biological samples were being taken from my child, I don't think I'd want to do that while they're so young.  I think I just would be more hesitant to have my baby's samples or specimens collected for a study. I'd wanna really look at all the different, the reasons for each type of sample collection. A swab I think I'd be a little more comfortable with than any of the others.  Honestly, I wouldn’t feel comfortable with you at all taking samples from my baby. From myself, maybe certain swabs from areas.  Just my child being touched in areas that they didn’t ask for. They didn’t ask to join this. I don’t know, I just feel it would make me super uncomfortable to bring my child somewhere to say, oh, you have to do this. We’re getting an incentive. I wouldn’t. That’s not worth anything for me unless I really had to take my child to the doctor and they had to get something for their health. I don’t think I can bring myself to do that for research. It doesn’t make me comfortable in that way.  I just don’t want to do that to my baby. That’s all. |
| **Concerns about data transparency and security** | I would like to know what all that would be done with these samples.  I would feel comfortable providing samples that are outer body, if that makes sense like swabs, urine samples. But I don’t think I would ever be comfortable sharing blood samples for genetic testing. I don’t think I’m prepared for that. That’s for myself. For the child, I don’t think I would be comfortable sharing any of it. Any of the testing.  I’d be comfortable with providing swabs for that [microbiome analysis] and non-invasive ones are okay. But you mentioned something about genetic material. So that would be something of concern to me. I want to know how is that material used? How is it stored? Would it be discarded? The confidentiality around genetic data?  I think it would take a lot for me to be comfortable with any swabs or et cetera for both myself and my baby. Partially, I think it’s different when it’s a public institution funded study versus private, but it’s so easy to have information, not be private or have security risks these days and also have it initially be for one purpose and then used for another. I would just be very wary of what it means if you were able to  get DNA blood information. |
